# Early Sagittal Shape of the Spine Predicts Scoliosis Development in a Syndromic (22q11.2DS) Population: A Prospective Longitudinal Study

**DOI:** 10.2106/JBJS.23.01096

**Published:** 2024-10-22

**Authors:** Steven de Reuver, Jelle F. Homans, Michiel L. Houben, Tom P.C. Schlösser, Keita Ito, Moyo C. Kruyt, René M. Castelein

**Affiliations:** 1 Department of Orthopaedic Surgery, University Medical Center Utrecht, Utrecht, The Netherlands; 2 Department of Pediatrics, University Medical Center Utrecht, Utrecht, The Netherlands; 3 Orthopaedic Biomechanics, Department of Biomedical Engineering, Eindhoven University of Technology, Eindhoven, The Netherlands; 4 Department of Developmental BioEngineering, University of Twente, Enschede, The Netherlands

## Abstract

**Background:**

Scoliosis is a deformation of the spine and trunk that, in its more severe forms, creates a life-long burden of disease and requires intensive treatment. For its most prevalent form, adolescent idiopathic scoliosis, no underlying condition can be defined, and the pathomechanism appears to be multifactorial; however, it has been suggested that the biomechanics of the spine play a role. For nonidiopathic scoliosis, underlying conditions can be recognized, but what drives the deformity remains unclear. In this study, we examined the early sagittal shape of the spine before the onset of scoliosis in a population with 22q11.2 deletion syndrome (22q11.2DS). This cohort was chosen since children with this syndrome have an approximately 50% chance of developing scoliosis that shares certain characteristics with idiopathic scoliosis, namely, age of onset, curve morphology, and rate of progression.

**Methods:**

This prospective cohort study included patients with 22q11.2DS who were followed with the use of spinal radiographs during adolescent growth. All of the children, who initially had no scoliosis while still skeletally immature (Risser stages 0 and 1), were followed at 2-year intervals until they reached skeletal maturity (Risser stages 3 to 5). We assessed the segment of the spine that has previously been shown to be rotationally unstable, the posteriorly inclined segment, to determine if it was predictive of later scoliosis development. For quantification, the area of the “posteriorly inclined triangle” (PIT), a previously described parameter that integrates both the inclination and length of the at-risk segment, was measured.

**Results:**

Of the 50 children who initially had no scoliosis (mean age at inclusion, 10.7 ± 1.7 years; mean follow-up, 4.8 ± 1.6 years), 24 (48%) developed scoliosis. Patients with an above-average PIT area (>60 cm^2^) at inclusion showed a relative risk of 2.55 for scoliosis development (95% confidence interval [CI]:1.22 to 5.34). PIT inclination was correlated with curve type: a taller and steeper hypotenuse predicted later thoracic scoliosis, while a shorter and less steep inclination predicted the development of (thoraco)lumbar scoliosis.

**Conclusions:**

This prospective study identified the pre-scoliotic sagittal shape of the spine as a risk factor for the later development of scoliosis in the population of children with 22q11.2DS.

**Level of Evidence:**

Prognostic Level II. See Instructions for Authors for a complete description of levels of evidence.

Scoliosis is a deformation of the spine and trunk that, in its more severe forms, creates a life-long burden of disease and requires intensive treatment. The cause of most types of scoliosis is unknown, even if an underlying disorder can be defined^1^. In congenital scoliosis, malformation and abnormal growth of the vertebrae can understandably lead to a progressive malformation of the spine^2^. In other types of scoliosis with a “known” origin (e.g., a syndromic or neuromuscular disease), the underlying condition does not explain the pathomechanism of the disorder. At the other end of the spectrum, with idiopathic scoliosis (the most prevalent form), not even an underlying pathology can be defined, and the cause has remained elusive and presumably multifactorial^1^. Previous studies have demonstrated that the human spine is rotationally less stable than other spines in nature since it has segments of vertebrae that are backwardly inclined and therefore subject to dorsal shear forces, suggesting that the sagittal shape of the spine has a role in the initiation of scoliosis^3-12^. To prove this concept in patients with idiopathic scoliosis would require prospective studies with use of ionizing imaging (such as radiographs) in a very large, healthy population, which would make the studies very difficult to carry out both ethically and practically. We prospectively studied the differences in the sagittal shape of the preadolescent and nonscoliotic spine in patients with 22q11.2 deletion syndrome (22q11.2DS). Patients with this syndrome have a variety of manifestations in many organ systems, but approximately 50% of the patients also develop scoliosis^13^. This syndromal scoliosis develops in spines without anatomical anomalies and has been shown to somewhat resemble adolescent idiopathic scoliosis (AIS) in terms of curve morphology and age of onset and progression rate^14^.

In this study, the posteriorly inclined segment in the sagittal profile of the spine was hypothesized to be a risk factor for scoliosis development. In order to study this hypothesis, the length and tilt angle of the posteriorly inclined segment were analyzed on sagittal standing full-spine radiographs that were made before the onset of scoliosis. To capture both the length and the tilt angle, we developed the concept of the “posteriorly inclined triangle” (PIT) (Fig. 1). This triangle can have different shapes depending on the length and inclination of the hypotenuse, but its surface area is an expression of the overall magnitude of the posteriorly directed, destabilizing vectors. This concept was first explored in a pilot study that included sample size calculations that we used in the current study^15^. The aim of the present study was to evaluate whether the sagittal shape of the spine before the onset of scoliosis can predict later scoliosis development in the population of patients with 22q11.2DS. We hypothesized that the children who ultimately develop scoliosis have more posteriorly directed, destabilizing vectors acting on their spine, as expressed by a larger PIT area. In addition, we expected that the shape of the initial triangle (the inclination of the hypotenuse) determines which vertebrae are at risk for rotating into scoliosis, and is thus related to the ultimate type of scoliotic curve that develops.

**Fig. 1:**
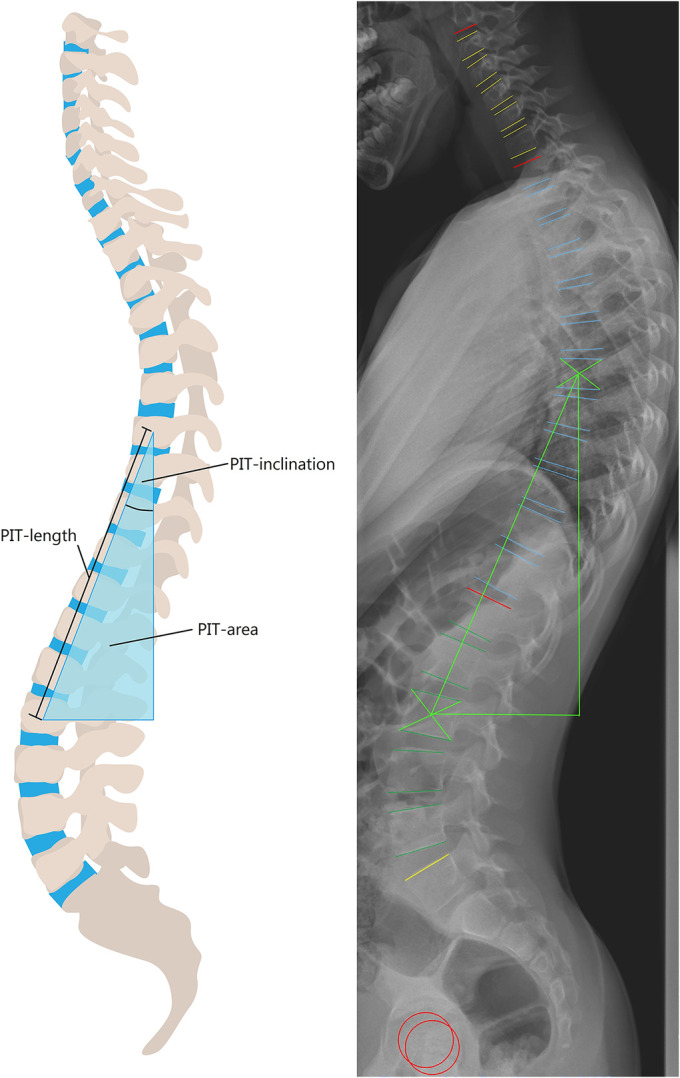
*Left:* A schematic view of the sagittal spine showing the PIT area, defined as the right-angled triangle between the centroids of the most cranial and most caudal posteriorly inclined vertebral bodies. *Right:* The analysis of the lateral radiograph with use of Surgimap software. The vertebral body end plates and femoral heads were semiautomatically segmented. The PIT (bright green) was automatically generated between the centroids of the most cranial and caudal posteriorly inclined vertebrae, which had also been automatically generated, and its area was measured.

## Materials and Methods

### Study Population

This prospective cohort study was conducted according to the STROBE (STrengthening the Reporting of OBservational studies in Epidemiology) guidelines for observational studies^16^. The University Medical Center Utrecht functions as the national referral center in The Netherlands for patients with 22q11.2DS; these patients are followed at regular multidisciplinary outpatient clinic visits from first diagnosis until adulthood. All of the caregivers are asked for broad consent to anonymously use data from the patient charts for research. From the age of 6 years, all patients with 22q11.2DS are screened for orthopaedic manifestations of scoliosis with radiographs (including anterioposterior and lateral full-spine views) once every 2 years. The institutional review board approved an exemption from individual informed consent for this study. We aimed to include 50 consecutive eligible patients aged 8 to 13 years who were skeletally immature (Risser stages 0 and 1) and had no scoliosis at first presentation (Cobb angle of <10°), as shown on their standing coronal full-spine radiographs^17,18^. We followed patients at least once every 2 years until they reached skeletal maturity (Risser stages 3 to 5). We excluded patients with additional genetic syndromes, congenital vertebral anomalies, or other orthopaedic manifestations influencing the spine or posture, as well as those who were undergoing growth hormone therapy and those who had undergone spine surgery; patients with insufficient radiographic examinations also were excluded.

### The PIT

All of the radiographic measurements were performed with Surgimap imaging software (version 2.3.2.1; Nemaris), which has been validated for measuring spinopelvic parameters^19^. A single trained observer blinded to the outcome analyzed the freestanding lateral full-spine radiographs, which had been made in a standardized manner, of every patient at inclusion. At that time, all of the patients were skeletally immature, and those with scoliosis on the coronal radiograph were excluded. First, the observer utilized Surgimap’s Spinal Wizard software to segment both femoral heads and all vertebral body end plates; manual adjustments were made where necessary (Fig. 1). Second, the software automatically calculated the inclination of each vertebral body based on the upper end plate in the sagittal plane, and the centroids of the most cranial and most caudal posteriorly inclined vertebrae were annotated. Third, the right-angled triangle between these 2 points and the vertical was drawn, and 3 parameters were determined: (1) the PIT area was calculated with the basic formula formula for the area of a triangle, 0.5 × width × height, (2) the PIT length was calculated as the length of the hypotenuse, and (3) the PIT inclination was calculated as the angle between the hypotenuse and the vertical (Fig. 1). Finally, the pelvic incidence^20^ and the length of the spine from T1 to S1 were measured, also in the sagittal plane, using the same Surgimap software. The T1-S1 spinal length was used to normalize the PIT area to account for absolute body size. This was done by calculating the ratio between the T1-S1 length of each patient and the study’s population mean, with no distinction between sexes since the T1-S1 length was not significantly different between boys and girls. Using this ratio allowed the PIT area and the PIT length to be normalized for the patient’s size but still retain their physical units (cm and cm^2^).

### End of Follow-up

To determine the presence or absence of scoliosis, the patient had to be skeletally mature (Risser stage ≥3) and there had to have been ≥2 years of follow-up. The most recent freestanding posteroanterior full-spine radiograph was analyzed. We determined the presence and curve size of scoliosis (Cobb angle of ≥10°), the level of the apex and the corresponding curve type, and whether the curve was primary thoracic or primary (thoraco)lumbar according to the Scoliosis Research Society guidelines^21^.

### Statistical Analysis

Based on an earlier pilot study, we identified a 1.5-fold difference in magnitude of the PIT area between those who would and would not develop scoliosis^15^. Using the mean and standard deviation of the PIT area from that study (59 versus 43 cm^2^ with a standard deviation of 20 cm^2^), we concluded that we would need 25 patients in each group in order to have enough statistical power to compare 2 means (with an independent-sample t test, a 2-sided alpha of 5%, and 80% power) with a sampling ratio of 1:1 (i.e., equal groups)^22^. Given that approximately 50% of patients with 22q11.2DS develop scoliosis, the required sample size was 50 participants^15,22^. We gathered data regarding the baseline characteristics, including sex and age, and follow-up interval. The normalized PIT area, the normalized PIT length, the PIT inclination, and the pelvic incidence showed a normal distribution, as tested with quantile-quantile (QQ) plots. Three groups based on the outcome of the last radiograph were compared: (1) patients with no scoliosis, (2) patients with primary thoracic scoliosis, and (3) patients with primary (thoraco)lumbar scoliosis. The difference between the sexes was analyzed with a Fisher exact test. The differences in age, follow-up, normalized PIT area, normalized PIT length, PIT inclination, and pelvic incidence among the 3 groups were analyzed with a 1-way analysis of variance (ANOVA) with a post hoc independent-sample t test and Bonferroni correction. First, the study population was split post hoc into 2 equal groups based on the mean PIT area at baseline (using a threshold of 60 cm^2^); scoliosis development in these 2 groups was compared, and the relative risk and 95% confidence interval (CI) were calculated. Second, the population was stratified into 6 ordinal groups according to the PIT area (0 up to 30, 30 up to 45, 45 up to 60, 60 up to 75, 75 up to 90, and ≥90 cm^2^), and the fraction of patients developing scoliosis in each group was calculated. The final analysis was a multivariable linear regression with PIT length and PIT inclination as predictors of the apex level of the eventual main scoliotic curve. Statistical analyses were performed with SPSS (version 27.0 for Windows; IBM). The significance level was 0.05.

## Results

### Patient Population

A total of 50 patients were consecutively included; 6 other patients were excluded (1 with an additional 7p21 duplication, 1 with a butterfly vertebra, 1 with a vertebral bar, 1 who was nonambulatory, and 2 who had malpositioned lateral radiographs). The mean age (and standard deviation) at inclusion was 10.7 ± 1.7 years, and the mean follow-up was 4.8 ± 1.6 years. Because patients who already had scoliosis (Cobb angle of ≥10°) were not included, the mean Cobb angle at inclusion was 5.0° (range, 0.0 to 9.7°); there was no significant difference between those who did and did not develop scoliosis during follow-up (p = 0.770). At the end of the follow-up, 8 patients were at Risser stage 3, 21 were at Risser stage 4, and 21 were at Risser stage 5. As expected, 24 (48%) of the 50 subjects had developed scoliosis: 12 had a primary thoracic curve, and 12 had a primary (thoraco)lumbar curve (Table I).

**TABLE I:** Preadolescent Clinical and Radiographic Parameters, Stratified by the Outcome at the End of Follow-up*

	Thoracic Scoliosis	(Thoraco)lumbar Scoliosis	No Scoliosis	P Value†
No.	12	12	26	
Boys	6 (50%)	6 (50%)	15 (58%)	0.87
Age at inclusion *(yr)*	10.6 ± 1.9	10.5 ± 2.0	10.9 ± 1.5	0.79
Follow-up *(yr)*	4.6 ± 1.5	5.4 ± 1.9	4.5 ± 1.5	0.26
Cobb angle *(deg)*	23.8 ± 13.7	16.3 ± 3.9	4.0 ± 3.6	<0.001
Preadolescent radiographic parameters at inclusion				
PIT area *(cm*^*2*^*)*	72 ± 26	75 ± 25	47 ± 20	<0.001‡
PIT length *(cm)*	24 ± 3	22 ± 2	18 ± 2	<0.001§
PIT inclination *(deg)*	15 ± 4	20 ± 5	16 ± 5	0.045#
Pelvic incidence *(deg)*	35 ± 5	44 ± 8	37 ± 7	0.006**

* The values are given as the count, with or without the percentage in parentheses, or as the mean± standard deviation.

† P values after Bonferoni correction are shown. In addition, post hoc tests with further Bonferroni corrections were performed, which showed the following.

‡ There was no difference in the normalized PIT area between thoracic and (thoraco)lumbar scoliosis, but both were larger than for patients with no scoliosis (p ≤ 0.01).

§ All pairwise differences in PIT length between groups were significant (p ≤ 0.047).

# The segment inclination angle was larger in (thoraco)lumbar scoliosis than in thoracic scoliosis (p = 0.048), but not significantly different from that in patients with no scoliosis.

** Pelvic incidence did not differ significantly between patients with thoracic scoliosis and those with no scoliosis, but it was significantly higher in patients with (thoraco)lumbar scoliosis than in both other groups (p ≤ 0.015).

### PIT Area

**Fig. 2:**
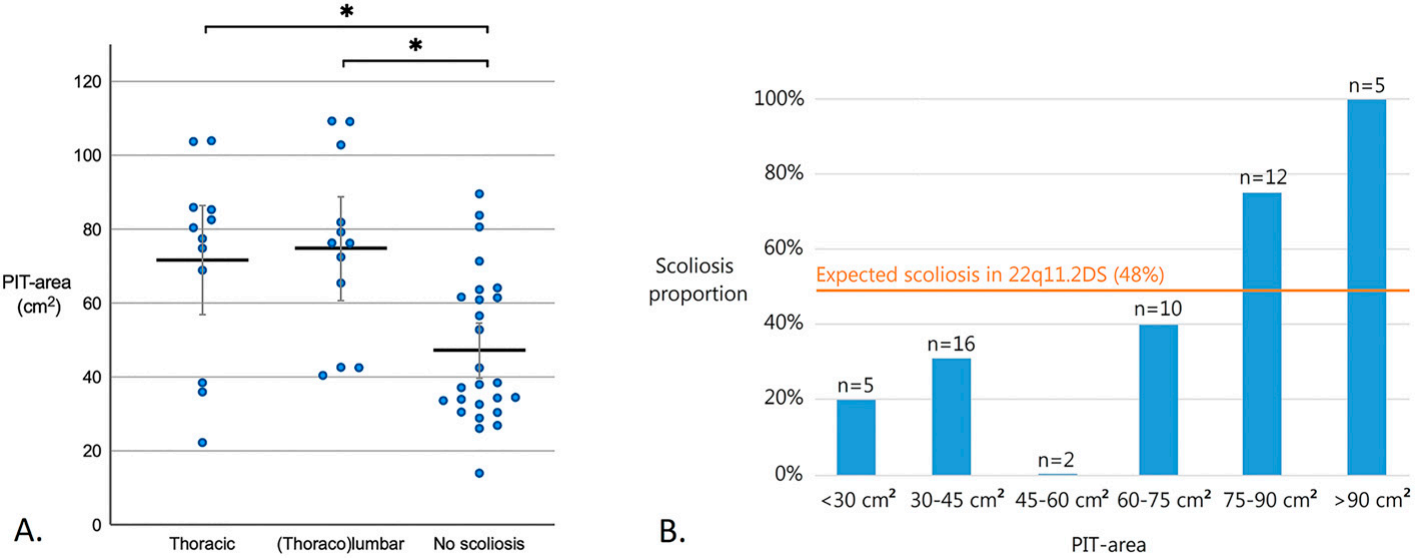
**Fig. 2-A** A plot of the normalized PIT area measured for each patient at inclusion, stratified by scoliosis type or no scoliosis at the end of follow-up. The horizontal bars indicate the mean PIT area, the vertical gray error bars indicate the 95% CI, and significant differences are indicated with an asterisk. **Fig. 2-B** The proportion of scoliosis cases, stratified by the normalized PIT area at study inclusion. Overall, 48% of the patients with 22q11.2DS developed scoliosis.

The area of the PIT in patients who would later develop scoliosis was 73 ± 25 cm^2^, more than 1.5 times the PIT area of 47 ± 20 cm^2^ in the patients who did not develop scoliosis. This effect was similar for both types of scoliosis, with a mean normalized PIT area of 72 ± 26 cm^2^ in patients with primary thoracic scoliosis and 75 ± 25 cm^2^ in those with primary (thoraco)lumbar scoliosis (Fig. 2, Table I). We observed an above-average PIT area of ≥60 cm^2^ at inclusion in 18 of 24 patients who eventually developed scoliosis, compared with 9 of 26 patients without scoliosis (i.e., relative risk, 2.55 [95% CI: 1.22 to 5.34]; Fig. 2). Comparing the PIT area subgroups, scoliosis developed in 20% of patients in the lowest group (PIT area, 0 to 30 cm^2^) and in 100% of patients in the highest group (PIT area, >90 cm^2^).

### Shape of the Posteriorly Inclined Segment

**Fig. 3:**
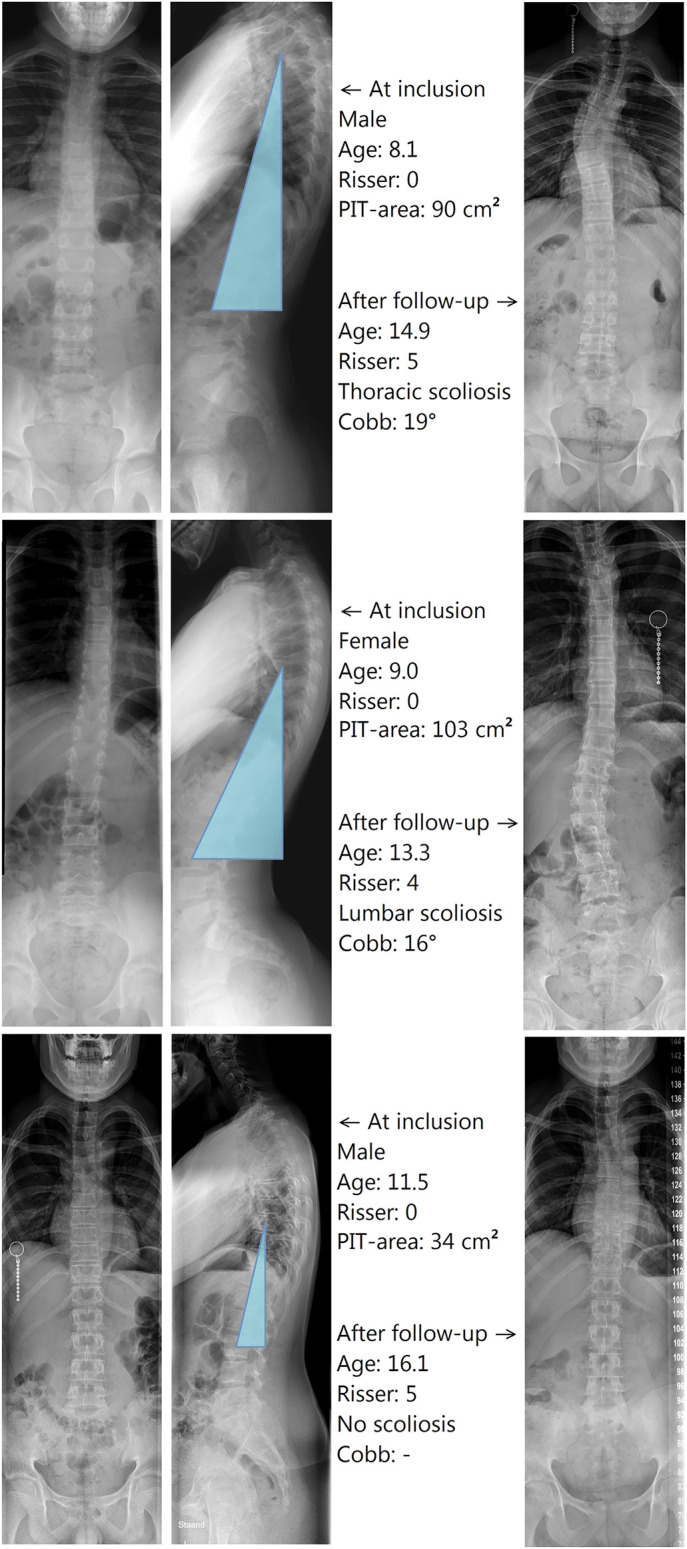
Radiographs of 3 study participants, demonstrating a larger PIT area before the development of scoliosis compared with the PIT area of patients who did not develop scoliosis. Note the tall and slender PIT in the patient with thoracic scoliosis compared with the wider and lower PIT in the patient with (thoraco)lumbar scoliosis.

**Fig. 4:**
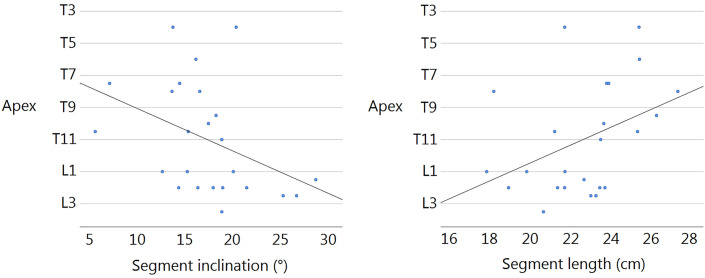
Scatterplots of the PIT inclination and PIT length versus the apex level of the main scoliotic curve for all of the patients who had developed scoliosis (n = 24). Multivariable linear regression analysis showed that, at inclusion, both normalized PIT inclination (r = −0.443, p = 0.020) and PIT length (r = 0.436, p = 0.022) were significant predictors of the apex level of the eventual main scoliotic curve.

The normalized hypotenuse length of the PIT was longest in patients with thoracic scoliosis (24 ± 3 cm), intermediate in patients with (thoraco)lumbar scoliosis (22 ± 2 cm), and shortest in patients with no scoliosis (18 ± 2 cm). The triangle was narrower but higher in those with thoracic scoliosis, with an inclination of 15° ± 4°, compared with those with (thoraco)lumbar scoliosis, with an inclination of 20° ± 5° (p = 0.048); however, both were not significantly different from those of patients with no scoliosis (inclination of 16° ± 5°) (Fig. 3, Table I). In a multivariable linear regression of the 24 scoliosis cases, both PIT length (r = 0.436, p = 0.022) and PIT inclination (r = −0.443, p = 0.020) were significant predictors of the apex level of the eventual main scoliotic curve (Fig. 4). Radiographs of patients with thoracic and lumbar scoliosis demonstrating the PIT area before the onset of scoliosis are shown in Figure 3.

### Pelvic Incidence

The pelvic incidence at inclusion in the overall group of patients who later developed scoliosis was not significantly different from that in the patients without scoliosis at the time of follow-up. The pelvic incidence was 35° ± 5° in patients who developed thoracic scoliosis, similar to those with no scoliosis (37° ± 7°), but it was significantly higher in those who developed a (thoraco)lumbar scoliosis (44° ± 8°) (Table I).

## Discussion

Sagittal plane spinal alignment has long been considered to play an important role in the development of scoliosis^3-13^. However, because all scoliosis research studies have been done on already established cases, it is impossible to distinguish if these differences in sagittal alignment are part of the cause of the deformity or rather its effect. To our knowledge, ours is the first study to prospectively demonstrate the role of the sagittal profile and dorsal shear forces in the later development of scoliosis, by utilizing a syndromic population that develops scoliosis in a high percentage of cases. The relevance to idiopathic scoliosis obviously remains to be established, but since the prevalence of scoliosis in the general population is only 2% to 4%, prospective studies using ionizing radiation (e.g., radiography) in a healthy pediatric population are not feasible, both ethically and practically^23^. Furthermore, biomechanical animal research cannot resolve this specific question because no animal model exists that reflects the unique spinopelvic sagittal alignment and biomechanical loading of the human spine^24^.

The posteriorly inclined spinal segment, quantified by the PIT area, strongly reflected the risk of ultimately developing scoliosis in this syndromic population. The PIT area was larger for patients who developed scoliosis than for those without scoliosis, but there was no difference between primary thoracic and (thoraco)lumbar curves. However, the PIT inclination did dictate the region of the spine in which scoliosis would develop: a more slender, higher, and vertical PIT area preceded thoracic curves, and a broader, lower, and more horizontal PIT area preceded (thoraco)lumbar curves. These distinct PIT shapes are essentially part of the known natural variation in sagittal spinal profile and pelvic shapes, as described in adults on the basis of the Roussouly types^25^. Indeed, our results show that a high pelvic incidence, which is associated with a higher sacral slope and thus a larger lumbar lordosis, is consequently also associated with a shorter and steeper posteriorly inclined segment, as well as the development of (thoraco)lumbar scoliosis. This confirms earlier observations regarding idiopathic scoliosis or adult degenerative scoliosis^12,20,26-28^.

Although the study was not powered for the analysis of patients with a PIT area above the average (60 cm^2^) compared with below the average, it showed a significant relative risk of 2.55 in the former group, stressing the strength of the PIT area as a risk factor. A small PIT area appeared to be protective against scoliosis development in patients with 22q11.2DS; however, the prevalence of scoliosis (20%) in this group was still well above the prevalence of scoliosis in the general population, underlining that the general increased risk of scoliosis in patients with 22q11.2DS may be caused by other risk factors. However, the risk of scoliosis development in this population was more than fourfold higher in those with a relatively large PIT area (>75 cm^2^) compared with patients with a smaller PIT area. Although the mean PIT area differences between cases and controls were very clear, there was much overlap between cases and controls, and no strict threshold for scoliosis development could be determined (Fig. 4). This implies that the PIT area is not suitable as a practical stand-alone tool for scoliosis risk assessment in specific individuals—it mainly provides a theoretical and conceptual argument in favor of a biomechanical component in the initiation of scoliosis. Currently, follow-up visits in our cohort of patients with 22q11.2DS take place at 2-year intervals until scoliosis develops, after which closer intervals are indicated. However, the outcome of this study may initiate a change in the protocol such that patients who have a greater PIT area and have not yet developed scoliosis are seen more frequently than every 2 years.

This study suggests that posterior inclination and dorsal shear forces are part of scoliosis etiology, in that the sagittal profile dictates which areas of the spine are rendered less stable in the horizontal (transverse) plane^6,29^. Of course, every human being in the general population has a posteriorly inclined segment, but not all develop scoliosis. It appears that a larger PIT area predisposes an individual to the development of scoliosis, but additional “triggers,” or enabling circumstances, are needed. Whether scoliosis actually occurs depends on the balance between the rotation-inducing forces and the stabilizers of the spine, mainly the intervertebral discs. A vulnerable period occurs when the body is rapidly increasing in size and weight while the intervertebral disc may still be in its own process of maturation (i.e., during the adolescent growth spurt)^30^. Interestingly, the increased risk of developing scoliosis in patients with 22q11.2DS cannot be explained by an increased dorsal inclination itself, as children from the general population (age 8 to 13 years) have a mean PIT area that is even slightly greater (as indicated by unpublished data derived from a previously investigated cohort). This indicates that important additional factors that cause the high scoliosis risk in patients with 22q11.2DS still remain to be identified. This population, which is under close medical scrutiny at our institution, has a scoliosis prevalence of approximately 50% compared with 2% to 4% in the general population, allowing for a prospective analysis of the role of the sagittal profile in the later development of scoliosis^1,13^. Obviously, scoliosis in this population is different from idiopathic scoliosis, although they both develop during growth in an anatomically normal and initially straight spine and share certain characteristics^14,31^. Extrapolation to the general population should obviously be done with caution.

### Conclusions

This prospective cohort study identified the magnitude of overall dorsal inclination (length combined with inclination angle) as a risk factor for the development of scoliosis in a syndromic population. The initial preadolescent sagittal spinal profile was shown to differ between patients who do and do not eventually develop scoliosis during adolescence. Furthermore, PIT inclination was shown to determine the type of scoliosis: a higher and narrower triangle preceded the development of thoracic scoliosis, and a broader and lower triangle preceded (thoraco)lumbar scoliosis. This substantiates that an important biomechanical component that is related to differences in individual spinal shape during growth is a risk factor for scoliosis development in this population of patients with 22q11.2DS.
